# Seroprevalence of *Borrelia burgdorferi* in Stray Dogs from Southern Italy

**DOI:** 10.3390/microorganisms8111688

**Published:** 2020-10-30

**Authors:** Paola Galluzzo, Francesca Grippi, Santina Di Bella, Francesco Santangelo, Sonia Sciortino, Alessandra Castiglia, Carmela Sciacca, Maria Arnone, Rosa Alduina, Giuseppina Chiarenza

**Affiliations:** 1 Istituto Zooprofilattico Sperimentale della Sicilia “A. Mirri”, via G. Marinuzzi 3, 90129 Palermo, Italy; paola.galluzzo@izssicilia.it (P.G.); sonia.sciortino@izssicilia.it (S.S.); alessandracastiglia1@gmail.com (A.C.); sciacca.carmela@gmail.com (C.S.); maria.arnone73@gmail.com (M.A.); giuseppina.chiarenza@izssicilia.it (G.C.); 2 Dipartimento Scienze e Tecnologie Biologiche, Chimiche e Farmaceutiche, Viale delle Scienze, University of Palermo, 90133 Palermo, Italy; valeria.alduina@unipa.it; 3 U.O. Igiene Urbana ASP Palermo presso Canile Municipale, Piazza Tiro a Segno 5, 90141 Palermo, Italy; franco.santangelo@alice.it

**Keywords:** *Borrelia burgdorferi*, seroprevalence, stray dogs, IFA, *ospA* gene

## Abstract

*Borrelia burgdorferi* is a bacterial pathogen transmitted by *Ixodes* ticks and is responsible for Lyme disease in both humans and dogs. The aim of this work was to evaluate *B. burgdorferi* diffusion among stray dogs in Palermo (Sicily, Italy) by serological methods in order to study the risk factors associated with the infection. Serum and blood samples of 316 dogs were collected from a shelter in Palermo, and were analyzed for the presence of antibodies against *B. burgdorferi* by indirect immunofluorescence assay (IFA), and of the *ospA* gene by real-time PCR, respectively. Seventeen sera (5.4%) were positive for the antibodies via IFA and one blood (0.3%) for *ospA* via real time PCR. On the basis of serological results, the evaluation of the potential risk factors (sex, age, breed and coat color) was carried out. The multivariate analysis indicated that male sex is a factor significantly associated with *B. burgdorferi* seropositivity. This study confirms that male dogs have a higher risk of developing the disease than females, and represents the first investigation on the spread of *B. burgdorferi* among stray dogs in Sicily.

## 1. Introduction

*Borrelia burgdorferi* is a spirochete causing Lyme disease, mainly in humans and dogs. In humans, Lyme borreliosis is characterized by an early set of skin-related and flu-like symptoms, and in absence of treatment, may be followed by arthritic or neurologic complications [1,2]. In Italy, the first case of Lyme borreliosis was recorded in northern Italy in 1983 [3]; afterwards, cases were observed in Sicily [4] and other regions [5,6,7,8]. At present, the epidemiology and spatial distribution of Lyme borreliosis cases are poorly studied in Italy, and the regional trend of disease incidence has not been described [9].

*B. burgdorferi* is usually transmitted by *Ixodes* spp. ticks, with *I. ricinus* known as the main vector in Europe. These arthropods are three-host ticks that acquire spirochetes during a blood meal from rodents, representing the main reservoirs, and can then transmit infection, as nymphs or adults [10], to humans, which are inadvertent hosts, and to other hosts that are not effective reservoirs for *B. burgdorferi.* In total, 32 vertebrate reservoir host species have been identified in Europe [11,12].

The disease is zoonotic and largely circulates within reservoir populations of wild animals, only relatively occasionally infecting humans. Among pet animals, dogs have been identified as a competent reservoir for *B. burgdorferi* [13]. Since systematic and randomized serological surveys are difficult and complex in human populations, the dog has been proposed as the ‘sentinel animal’ for the detection of emerging risk areas of Lyme disease [14,15,16,17]. The canine disease is often mild, without specific clinical manifestations, commonly characterized by lameness, fever, anorexia, lethargy and lymphadenopathy. In some cases, the disease can be severe, with arthritis and neurologic dysfunction [18,19,20,21]. Dogs, as wildlife hosts, can act as carriers of infected ticks from natural to human settings [22,23]. For this reason, dogs represent good indicators of the exposure of humans to infected ticks, since they largely share the same environment and visit the same outdoor areas. Studies have suggested that canine seroprevalence of *B. burgdorferi* greater than 5% may be a sensitive but non-specific marker of risk for humans [24,25].

The presence of tick vectors, and in particular the number and the frequency of infected ticks, is a prerequisite for infection and disease risk increases. Ticks are relatively more abundant in woodland, heath and moorland habitats, but can be found even in urban parks where hosts are plentiful [12]. Stray dogs pose a significant threat to human health due to their role as vectors of zoonotic diseases and parasites. Stray and sheltered dog populations are a known, yet underestimated, problem in several countries located in the northern and southern hemispheres, as well as in the studied area [26]. Therefore, the purpose of the present study was to evaluate the spread of *B. burgdorferi* in Palermo, southern Italy, among stray dogs, by using both serological and molecular methods.

## 2. Materials and Methods

### 2.1. Study Design

During the period January–September 2018, 316 serum samples and their corresponding 316 blood samples were collected from stray dogs present in the shelter of Palermo (Sicily, Italy). The sample size was determined using *Winepi* software considering an expected prevalence of 50%, with 5% precision at the 95% confidence level (as no other epidemiological data were available). According to *Winepi*, the minimum number of required samples was 285 animals.

The dogs analyzed were randomly chosen among the 1100 dogs that passed through the shelter during the studied period. Veterinarians reported no overt symptoms of borreliosis in the dogs that were considered clinically healthy.

### 2.2. Serological Test

Blood samples were collected into a 10 mL vacuum tube, stored in a refrigerated bag and conferred to the laboratories of Istituto Zooprofilattico Sperimentale of Sicily. Antibodies to *B. burgdorferi* were detected by the commercial test Canine Borreliosis IgG IFA (FULLER Laboratories, Fullerton, CA, USA) according to the manufacturer’s instructions. The results were visualized using standard fluorescence microscopy, where a positive reaction was seen as sharply defined apple-green such as was seen in the negative control well. All sera with titres ≥1:64 were considered positive (Table 1).

### 2.3. DNA Extraction and Real-Time PCR

DNA was extracted from canine whole blood using the commercial kit MagMAX CORE (Thermo Fisher Scientific, Monza, Italy), following the manufacturer’s protocol, and stored at −20 °C. Real-time PCR was performed using the CFX96 Touch Real-Time PCR Detection System (Bio-Rad, Hercules, CA, USA), amplifying a 102-base pair product of the *ospA* gene encoding for an outer surface protein [27]. The sample mix included a TaqMan probe, which anneals specifically to a 24-nucleotide sequence in the amplified product. About 200 ng/µL of DNA was added to a 20 µL mix containing 1X of SSo advanced Universal Probes Supermix (Bio-Rad) and 0.5 µL of each primer Bor_OspA F 5′-AATATTTATTGGTAGGTCTAA-3′ (10 µM), Bor_OspA R 5′-CACCAGGCAAATCTACTGA-3′ (10 µM) and TaqMan probe Bor_OspA TM FAM-TTAATAGCATGYAAGCAAAATGTTAGCA-BHQ1 (10 µM). The instrument program consisted of an initial denaturation of 3 min at 95 °C followed by 40 cycles of amplification, which included a denaturation step at 95 °C for 10 s and an annealing/extension step at 60 °C for 30 s.

### 2.4. Statistical Analysis

The statistical analysis was performed via multiple logistic regression models on the positive samples showing titres ≥1:128. The significant variables were identified using the maximum likelihood estimates. To assess the risk factors, corresponding *p*-values and odds ratios (OR) for the examined variables were evaluated, with a 95% confidence interval (95% CI). The mathematical modeling was performed using the SAS/STAT software version 9.4 (SAS Institute Inc., Cary, NC, USA).

The dogs were compared with respect to age (puppy ≤1 year, adult from 1 to 7 years and senior ≥7 years), sex (male or female), breed (purebred or mixed breed) and coat color (fair, dark or mixed color). The cut-off points were selected considering the average characteristics of the analyzed dogs.

### 2.5. Ethical Statement

The study did not involve any animal experiment. Only sample collection from naturally infected dogs was carried out consisting of a single blood draw per dog. This was needed for the laboratory analyses and did not involve any suffering of the sampled animals. This study was conducted as part of the IZS SI 11/16 RC research project entitled “Indagine sulla diffusione di Borreliosi e Bartonellosi in canili del territorio regionale e potenziali rischi zoonosici correlati” approved by the Italian Ministry of Health on 9th August 2017 (DGSAF-00189113-P-09/08/2017).

## 3. Results

### 3.1. Animal Demographics

A total of 316 (161 male and 155 female) dogs were tested. The dogs were divided into three groups based on age—108 puppies (less than 1 year old), 150 adults (between 1 and 7 years old), 58 senior (over 7 years old). Most dogs involved in the study were mixed breed (177 dogs) and 139 were abandoned purebreeds. In relation to the coat color, 104 dogs had a fair coat, 122 a dark coat and 90 a mixed color coat.

### 3.2. Serological Tests

Out of 316 sera samples, 25 had antibodies against *B. burgdorferi*, 8 with a titer of 1:64, 10 with titer of 1:128 and 7 with titer of 1:256 (Table 1). Positive sera at dilutions greater than or equal to 1:128 were taken into account to avoid false positives due possible cross-reactions with other *Borrelia* strains, as already demonstrated [28,29]. Thus, the seroprevalence was 5.4%.

### 3.3. Molecular Analysis

The evaluation of *B. burgdorferi* DNA presence in the whole blood of all the 316 dogs carried out by real-time PCR showed, to our surprise, that only one dog (0.3%) contained *B. burgdorferi* DNA. The serum of this dog tested negative via IFA.

### 3.4. Risk Factors Analysis

The obtained results were examined by multivariate analysis in order to determine the correlation, if any, between IFA results and four different variables: age, sex, breed and coat color (Table 2). The multivariate analysis showed that only the sex variable was significantly related to *B. burgdorferi* seropositivity (χ2= 6.357; *p* < 0.0117). Male dogs had a risk about five-times greater of developing the disease compared to females. In fact, the seroprevalence in male dogs was 8.9%, while in females it was 1.9%. No correlation was found for other variables.

## 4. Discussions and Conclusions

Lyme disease is a multisystemic inflammatory disorder caused by an immune response to the spirochete *B. burgdorferi*, which is transmitted by ticks, primarily by *Ixodes ricinus* in western Europe. Lyme disease’s eco-epidemiology is complex and depends on environmental features, such as tick development and survival, abundance and type of spirochete reservoirs, hosts that contribute to the spread of ticks, and human exposure to tick-bites. The epidemiology and spatial distribution of the Lyme disease cases are poorly studied in Italy, and the regional trend of disease incidence has not been described [9]. The disease is probably underdiagnosed and underreported, likely because only about 5–10% of dogs exposed to infected ticks develop clinical borreliosis [30], and because many human infections are not recognized [2].

Dogs are often the first to be exposed to microbes and contaminants that can cause illness in people. In particular, dogs are effective sentinels for vector-borne diseases, such as Lyme disease, since they can be easily exposed to ticks that harbor pathogens in the long grass and shrub land. The prevalence of anti-*Borrelia* antibodies in dogs may reflect the prevalence of the pathogen and the frequency of tick bites in the environment.

There are not many references regarding *B. burgdorferi* seroprevalence in stray dogs, although dogs housed in shelters are a high-risk population, carrying or spreading a variety of important diseases to both animals and humans, due to the conditions of antigenic life span in shelters, high population density, stress and exposure to rodents, and arthropod vectors [31].

The seroprevalence found in the stray canine population of Palermo (5.4%) is similar to that of stray dogs reported in Spain (8.8%), while our data are in disagreement with published data collected with a seroprevalence of 0.6%, which was found in Sofia and in Romania [32,33], and no dog was found positive for *B. burgdorferi* in Korea, in Algiers, in Mauritius, in Malasya, or in Jordan [34,35,36,37,38]. Our findings are similar also to those reported in other geographical areas on other types of dogs (owned, hunting, deceased): 5% in Croatia [39], 6.2% in Bulgaria [40], 6.5% in Romania [41], and 4.9% in Germany [42]. A higher seroprevalence of *B. burgdorferi* among dogs was reported in the Czech Republic (10.3%) [43], in the Netherlands (17%) [44] and in Serbia (22.3%) [45]. On the other hand, lower seroprevalence in apparently healthy dogs was detected in Portugal (0.2%), Spain (0.4%), France (1.1%) and Latvia (2.5%) [46,47,48,49].

In southern Italy a seroprevalence of 0.3% in hunting dogs [50] was reported, and in Tuscany a seroprevalence of 1.5%, with a lower rate in urban dogs than the rural ones, was reported [2].

Different canine seroprevalences against *B. burgdorferi* throughout Europe could be dependent on the geographical area, season variability and test employed [30].

Data concerning the prevalence of *B. burgdorferi* in stray dogs in other regions are lacking and were often outdated; therefore, it is not possible to affirm if the results of this study are in agreement with the prevalence in dogs living in other Italian regions, or in the same regions but in different periods.

*I. ricinus* (*Acarina: Ixodidae*) is the most widespread hard tick species in Italy, and is largely distributed in the area of the present study (Sicily, Italy) [51].

Molecular investigation, by real-time PCR, showed the presence of bacterial DNA only in one canine sample, which appeared healthy. Thus, this result demonstrates the low diffusion of the pathogen into the kennel at the time of the sampling, even if technical limitations, due to the low presence of genomic copies detectable in small volumes of blood, cannot be ruled out [52]. The detection of *B. burgdorferi* genomic DNA in blood by PCR occurs more easily in patients with early Lyme disease [53]. This could explain why the sample that we found to be positive via PCR was negative in the serological tests that detected the presence of IgG [52].

The greater risk of the male dogs developing the disease compared to females is probably dependent upon the longer life of males in the stray period. This result is in accordance with a previous study that indicated male dogs are more predisposed to *B. burgdorferi* exposure due to a greater wandering than females [28].

The other variables analyzed in this work seem to have no significant differences, even if other authors have reported a greater degree of seropositivity in dogs aged between the first and second year of life [16,28,44,54,55]. Regarding the coat color, only one reference [56] showed significant differences in seropositivity related to a dark coat.

This study reports the first investigation on the spread of *B. burgdorferi* among stray dogs in Sicily; in addition, since dogs represent a sentinel animal for tick-borne infection, this study could be a starting baseline for future comparison in order to locate the foci of, and to monitor the changes in, the environmental risk of Lyme borreliosis.

## Figures and Tables

**Table 1:** Characteristics of dogs that showed antibodies against *B. burgdorferi*: titre and the four variables considered (sex, age, race and coat color).

Titres	Sex	Age	Race	Coat Color
1:64	Female	Senior	Mixed breed	Mixed color
		Puppy	Mixed breed	Mixed color
	Male	Puppy	Purebreed	Mixed color
		Puppy	Purebreed	Dark
		Adult	Purebreed	Mixed color
		Adult	Purebreed	Dark
		Senior	Mixed breed	Dark
		Senior	Mixed breed	Fair
1:128	Female	Puppy	Mixed breed	Mixed color
		Senior	Mixed breed	Fair
		Adult	Purebreed	Dark
	Male	Puppy	Mixed breed	Mixed color
		Adult	Mixed breed	Fair
		Senior	Mixed breed	Fair
		Senior	Mixed breed	Fair
		Senior	Mixed breed	Dark
		Senior	Mixed breed	Dark
		Adult	Purebreed	Fair
1:256	Male	Puppy	Mixed breed	Mixed color
		Puppy	Mixed breed	Mixed color
		Senior	Mixed breed	Mixed color
		Senior	Mixed breed	Dark
		Adult	Purebreed	Mixed color
		Adult	Purebreed	Mixed color
		Adult	Purebreed	Mixed color

**Table 2:** *Borrelia* seropositivity according to characteristics of dogs: percentage of positive, number of positive, odds ratio related 95% confidence intervals (95% CI); Reference (Ref.).

Factors	% pos	N° pos/all	OR (95% CI)
Age			
Puppy	3.7	4/108	0.314 (0.084–1.177)
Adult	4	6/150	0.405 (0.122–1.343)
Senior	12	7/58	Ref.
Sex			
Male	8.7	14/161	5.396 (1.456–20.005)
Female	1.9	3/155	Ref.
Breed			
Purebreed	3.5	5/139	Ref.
Mixed breed	6.7	12/177	2.522 (0.776–8.191)
Coat color			
Mixed color	8.8	8/90	Ref.
Fair	4.8	5/104	0.357 (0.104–1.231)
Dark	3.2	4/122	0.290 (0.080–1.043)
